# Machine Learning Enhanced Dynamic Response Modelling of Superelastic Shape Memory Alloy Wires

**DOI:** 10.3390/ma15010304

**Published:** 2022-01-01

**Authors:** Niklas Lenzen, Okyay Altay

**Affiliations:** Lehrstuhl für Baustatik und Baudynamik, Department of Civil Engineering, RWTH Aachen University, 52074 Aachen, Germany; lenzen@lbb.rwth-aachen.de

**Keywords:** machine learning, artificial neural networks, shape memory alloys, superelastic, parameter identification, constitutive model, thermodynamic parameters

## Abstract

Superelastic shape memory alloy (SMA) wires exhibit superb hysteretic energy dissipation and deformation capabilities. Therefore, they are increasingly used for the vibration control of civil engineering structures. The efficient design of SMA-based control devices requires accurate material models. However, the thermodynamically coupled SMA behavior is highly sensitive to strain rate. For an accurate modelling of the material behavior, a wide range of parameters needs to be determined by experiments, where the identification of thermodynamic parameters is particularly challenging due to required technical instruments and expert knowledge. For an efficient identification of thermodynamic parameters, this study proposes a machine-learning-based approach, which was specifically designed considering the dynamic SMA behavior. For this purpose, a feedforward artificial neural network (ANN) architecture was developed. For the generation of training data, a macroscopic constitutive SMA model was adapted considering strain rate effects. After training, the ANN can identify the searched model parameters from cyclic tensile stress–strain tests. The proposed approach is applied on superelastic SMA wires and validated by experiments.

## 1. Introduction

Shape memory alloys (SMAs) are superelastic two-phase polycrystal metals. During dynamic loading, repeated forward- and reverse-phase transitions occur allowing the material to dissipate energy. Besides this key property, SMAs exhibit also other unique characteristics, such as large deformation recovery, corrosion resistance, and low fatigue. Therefore, SMA-based vibration control devices have been a particular research field in civil engineering. Qiu and Zhu [1] presented a self-centering steel frame, which utilizes superelastic Ni-Ti wires within a SMA-based damper. Moreover, Liu et al. [2] proposed a base isolation system by incorporating springs made of superelastic SMA wires. Apart from this, Liang et al. [3] used cables composed of SMA wires to enhance the effectiveness of a friction sliding bearing. A review and detailed summary of the related applications can be found in [4,5,6]. SMAs are also being used in other dynamic systems, such as in aeronautic [7] and automotive [8] engineering.

For the design of SMA-based control devices, accurate constitutive models are required, whereas due to their numerical efficiency, macroscopic models are generally preferred. Furthermore, for an efficient heat transfer, most control devices incorporate SMAs as wires, due to which uniaxial models are particularly necessary. Brinson [9] and Auricchio and Sacco [10], among others, developed fundamental one-dimensional macroscopic models. Since then, several one-dimensional thermomechanically coupled constitutive models were designed, such as in [11,12]. Improved versions of these models are also proposed in [13,14]. For a detailed review on other modelling techniques, we refer the interested readers also to [15] and references therein.

A common characteristic of constitutive SMA models is that their accuracy relies on the utilized material parameters, which need to be identified by experiments. In case of SMA wires, uniaxial tensile tests are sufficient to obtain stress–strain relations. The parameter identification (PI) process of thermodynamic properties requires specific instruments and an expert knowledge-based data processing, such as thermomechanical analysis or more specifically the differential scanning calorimetry (DSC) [16,17]. A more important aspect is the fact that even with perfectly identified parameters, constitutive models require a supplementary tuning step for accurate response computations. Machine learning (ML) methods, particularly artificial neural networks (ANNs), provide efficient and versatile solutions, which can circumvent these challenges.

ANNs are capable of predicting highly nonlinear relations with any desired degree of accuracy according to the universal approximation theorem [18] and have already been used as black-box models for the mapping of constitutive relations, such as in [19,20]. In the field of SMAs, in their pioneering study, Ozbulut and Hurlebaus [21] proposed a neurofuzzy model, which predicts SMA responses from strain, strain rate, and temperature inputs. Although black-box models are efficient, they generally require a large set of representative experimental data.

In the context of PI, Huber and Tsakmakis [22] successfully implemented a feedforward ANN, which determines the parameters of a finite deformation viscoplasticity model. Furthermore, for superelastic SMAs exposed to quasistatic loading, Helm [23] developed a constitutive model and proposed a three-layered feedforward ANN architecture to identify the required model parameters from stress inputs. In this approach, similar to Huber and Tsakmakis, the ANN is trained by data, which are numerically generated by the model using parameters that are randomly sampled within a predefined space. Similarly, Henrickson et al. [24] trained an ANN to identify transformation temperatures of SMAs by using strain–temperature curves as inputs.

This paper proposes particularly for dynamic applications of superelastic SMA wires an ANN-based PI methodology, which considers the strain rate dependency of the material and focuses on the identification of thermodynamic parameters from stress–strain responses. Within the PI methodology, the identified parameters are already being tuned considering the constitutive model to allow accurate response computations.

The remainder of the paper is organized as follows: In Section 2, the approach is presented and implemented within the macroscopic modelling frame of Zhu and Zhang [12]. In Section 3, the approach is applied to SMA wires and validated by experiments. Finally, the conclusions of the study are drawn in Section 4.

## 2. Methodology

### 2.1. Superelastic SMA Response

SMAs are characterized by austenite (*A*) and martensite (*M*) phase states. Above the austenite transformation finish temperature $A_{f}$, the parent phase is austenite, and SMAs exhibit superelastic behavior. Hence, high mechanical stresses induce a forward-phase transformation (*AM*), and the SMA crystals reorient their atomic grid from body centered (B2) to a monoclinic (B19) lattice, which is more stable for high stress levels. Upon unloading, a reverse-phase transformation (*MA*) occurs, and the material returns to its original shape without residual deformation. During both forward- and reverse-phase transformations, SMAs exhibit a pseudoplastic deformation, which can be observed by stress plateaus in the stress–strain response. The *AM* transformation is an exothermic process. The lattice rearrangement causes internal heating, which is released to the environment via heat convection and conduction. By contrast, the *MA* transformation is a strongly endothermic reaction, in which the austenite formation is accompanied by a reduction in material temperature.

Dynamic loading patterns generally involve high strain rates, which impair the release of the heat generated within the forward transformation. This affects the material, such that the stress plateau slope increases since the austenite phase is energetically more stable for the increased temperature. Consequently, the reverse transformation is also initiated on higher critical stress levels affecting the stress–strain curve and the associated hysteretic energy dissipation.

**Figure 1:**
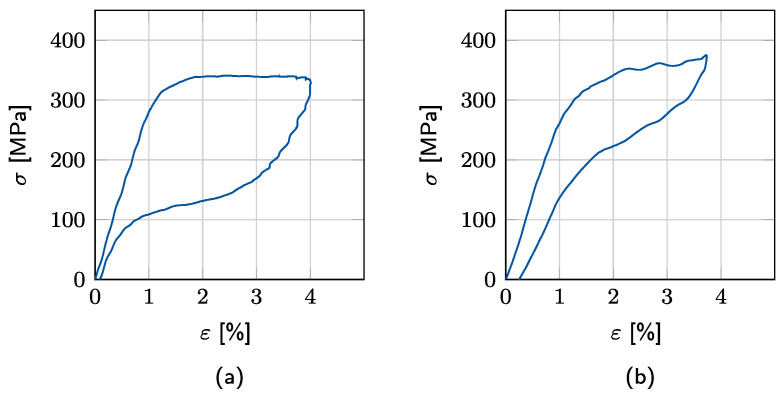
Cyclic tensile tests on an SMA wire. Strain rate amplitudes: (**a**) quasistatic ${\dot{\varepsilon }}_{a}=1.26$% s^−1^ and (**b**) dynamic ${\dot{\varepsilon }}_{b}=50.27$% s^−1^.

To illustrate the strain rate effects, experimentally determined stress–strain responses of an SMA wire are depicted in Figure 1. The alloy composition of the wire is Ni-55.8%-Ti-43.95%. The wire length and diameter are l=150 and d=0.2 mm, respectively. A pre-stress of $\sigma_{0}=134.9$ MPa is applied. The ambient temperature is around $T_{init}=296.2$ K (23.05 °C), whereas the austenite transformation finish temperature of the wire is $A_{f}=285.2$ K (12.05 °C). Accordingly, the SMA is expected to response superelastically. Two cyclic loading patterns are applied with the strain rate amplitudes of ${\dot{\varepsilon }}_{a}=1.26$% s^−1^ and ${\dot{\varepsilon }}_{b}=50.27$% s^−1^. The *AM* transformation plateau changes from horizontal to a steeper slope with the increasing strain rate as the generated heat cannot be released directly. High strain rates also reshape the *MA* transformation, which starts at a higher stress with a steeper slope.

### 2.2. Machine-Learning-Based Parameter Identification

**Figure 2:**
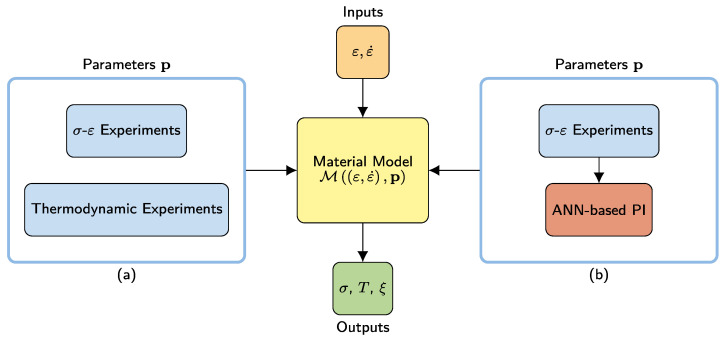
(**a**) Conventional experimental identification and (**b**) proposed machine-learning-based identification of thermodynamic parameters for use in constitutive modelling. The constitutive model is driven by the strain ε and strain rate $\dot{\varepsilon }$ time histories. Stress σ, temperature *T*, and the martensite volume fraction ξ time histories are computed as model outputs.

Macroscopic models of superelastic SMA wires generally compute the stress response σ, temperature *T*, and martensite volume fraction ξ from strain ε and strain rate $\dot{\varepsilon }$. Accordingly, such models can be represented as $M\left(\left(\varepsilon ,\dot{\varepsilon }\right),p\right)$, where the vectors ε and $\dot{\varepsilon }$ are time histories of strain and strain rate, respectively. The vector p contains model parameters, which need to be determined from experiments. Conventionally, as shown in Figure 2a, the stress–strain (σ-ε) experiments are conducted to investigate the cyclic tensile stress and strain response characteristics. Furthermore, thermodynamic experiments are required to investigate the model parameters representing the thermodynamic characteristics. Alternatively, in this study, as shown in Figure 2b, to reduce the experimental effort, an ML-based procedure is proposed for the identification of the thermodynamic parameters from stress–strain experiments by using an ANN. Different types of ML models could realize a better performance as well. However, in this study, ANNs are preferred considering their capability and efficiency in representing the extreme nonlinearities of SMAs.

**Figure 3:**
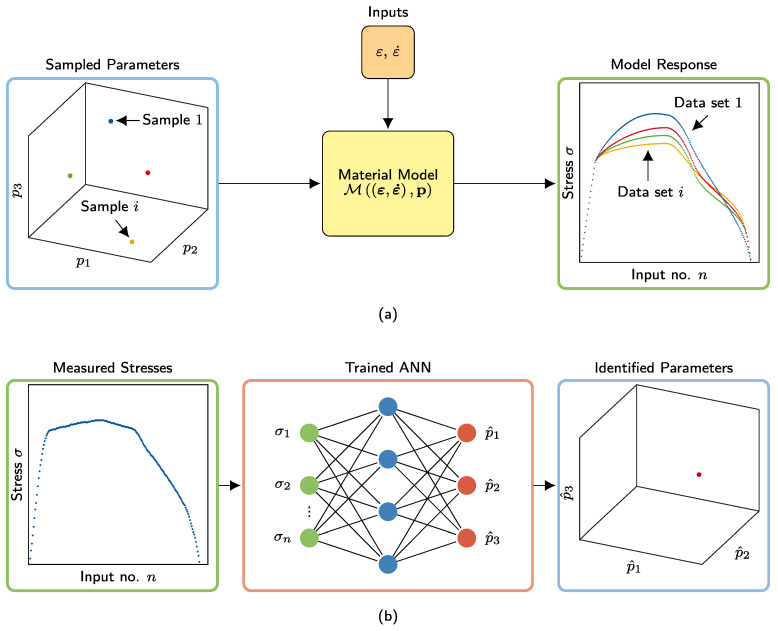
(**a**) Forward step of the proposed parameter identification procedure: Thermodynamic parameter samples $p_{i}$ are fed into the material model to produce *n* stress outputs. (**b**) Reverse step of the proposed parameter identification procedure: Trained ANN estimates searched thermodynamic parameters $\hat{p}$ from experimental stress–strain response.

The procedure consists of forward and reverse steps. As shown in Figure 3a, in the forward step, a constitutive SMA model generates training data for the ANN. In this study, due to its robustness and accuracy, the constitutive model by Zhu and Zhang [12] is chosen and adapted considering dynamic effects, as described in Section 2.3. Other models, such as by Auricchio and Sacco [11], could be implemented as well.

The searched thermodynamic parameters $p_{i},\forall i\in \left\{1:N\right\}$ are sampled from a predefined parameter space by the Latin hypercube sampling (LHS) method [25], where *N* is the number of samples. In Figure 3, as an example, three parameter types ($p_{i}^{⊤}={\left[p_{1}\;p_{2}\;p_{3}\right]}_{i}$) are searched. Accordingly, the parameter sampling space is three-dimensional here. At this point, it should be noted that, besides material parameters, during PI process, one should particularly consider those parameters as variable, which directly affect the reverse transformation, cf. Figure 1 and $A_{s,d}$ in Section 3.2. Each sampled parameter set $p_{i}$ is then fed separately into the constitutive model, such that the corresponding SMA stress response vector $\sigma_{i}$ is generated as the training data set $D=\{\left(\sigma_{i},p_{i}\right),\forall i\in \left\{1:N\right\}\}$. Each computation is conducted for one load cycle, which is represented by the strain $\varepsilon_{i}\in R^{n\times 1}$ and strain rate ${\dot{\varepsilon }}_{i}\in R^{n\times 1}$ time histories, where *n* is the number of time instants. Accordingly, each generated stress time history $\sigma_{i}\in R^{n\times 1}$ consists of loading $\sigma_{i}^{AM}$ and unloading $\sigma_{i}^{MA}$ paths.

As shown in Figure 3b, the reverse step is built as a feedforward ANN, in which the output $p^{\left(1\right)}$ of the first hidden layer and the output $p^{\left(l\right)}$ of the final layer read

(1) $$p^{\left(1\right)}=f^{\left(1\right)}\left(W^{(1)⊤}\sigma +b^{\left(1\right)}\right),\;p^{\left(l\right)}=\hat{p}=f^{\left(l\right)}\left(W^{(l)⊤}y^{\left(l-1\right)}+b^{\left(l\right)}\right),$$

where l−1 is the number of hidden layers, which depends on the complexity of the material model. Theoretically, one layer is enough according to the universal approximation theorem [18]. For constitutive models using a limited number of parameter types, shallow ANNs with 2–3 layers are suggested, as shown later in Section 3. In Equation (1), *f* is the activation function, W is the weight matrix, σ is the stress input vector, and b is the bias vector. The vector $\hat{p}$ contains thermodynamic parameters, which are predicted by the ANN. For the training, the mean squared error function is applied, which is optimized within the backpropagation algorithm by the Adam optimizer [26]. In total, 10% of the generated data D is spared for validation. The hyperbolic tangent activation function is used in the hidden layers, whereas the sigmoid function is used in the final layer to scale the outputs between 0 and 1. To avoid vanishing gradients, a batch normalization algorithm [27] is applied. Additionally, the dropout algorithm [28] is used after activation of the hidden layers to prevent the overfitting of the training data.

### 2.3. Constitutive Modelling of Superelastic SMA Wire Response

The constitutive material model by Zhu and Zhang [12] is based on the first and second law of thermodynamics. Similar to the work of Tanaka [29], the material model is strain-driven and uses the state variables temperature *T* and martensitic volume fraction ξ to compute the one-dimensional tensile stress behavior of SMA wires. In analogy to Sadjadpour and Bhattacharya [30], the Helmholtz free energy is computed per unit mass by

(2) $$\psi =\frac{E}{2\rho }\varepsilon_{el}^{2}+\frac{L}{T_{cr}}\left(T-T_{cr}\right)\xi -CT\ln\left(\frac{T}{T_{init}}\right),$$

where ρ is the density, $\varepsilon_{el}$ is the elastic strain, $T_{cr}$ and $T_{init}$ are the transformation and initial (ambient) temperatures, *E* is the Young’s modulus, *L* is the latent heat of the phase transition and *C* represents the specific heat. Here, the Young’s modulus is expressed according to Liang [31] and Sato and Tanaka [32] as a function of the martensite volume fraction to allow for a transition between the two phases as

(3) $$E\left(\xi \right)=E_{A}+\xi \left(E_{M}-E_{A}\right),$$

where $E_{A}$ and $E_{M}$ are constants representing the corresponding elastic moduli of austenite and martensite phases, respectively. The elastic $\varepsilon_{el}$ and inelastic $\varepsilon_{in}$ strains are expressed according to Brinson [9] and Auricchio and Sacco [10] as

(4) $$\varepsilon_{el}=\varepsilon -\varepsilon_{in},\;\varepsilon_{in}=\varepsilon_{l}\xi ,$$

where $\varepsilon_{l}$ is the maximum strain after a complete *AM* transformation with the martensite portion ξ=1. Furthermore, the heat equation is derived from the first law of thermodynamics as

(5) $$\begin{array}{cc} C\dot{T} & =-\frac{\partial \psi }{\partial \xi }\dot{\xi }+T\frac{\partial^{2}\psi }{\partial \varepsilon \partial T}\dot{\varepsilon }+T\frac{\partial^{2}\psi }{\partial T\partial \xi }\dot{\xi }-\frac{k}{V\rho }\left(T-T_{init}\right)\\ \; & =\frac{\sigma }{\rho }\varepsilon_{l}\dot{\xi }+L\dot{\xi }-\frac{k}{V\rho }\left(T-T_{init}\right), \end{array}$$

where *k* and *V* are the heat-transfer coefficient and the specimen volume, respectively. The stress response of the SMA wire is then computed by

(6) $$\sigma =\rho \frac{\partial \psi }{\partial \varepsilon }=E\varepsilon_{el}$$

**Figure 4:**
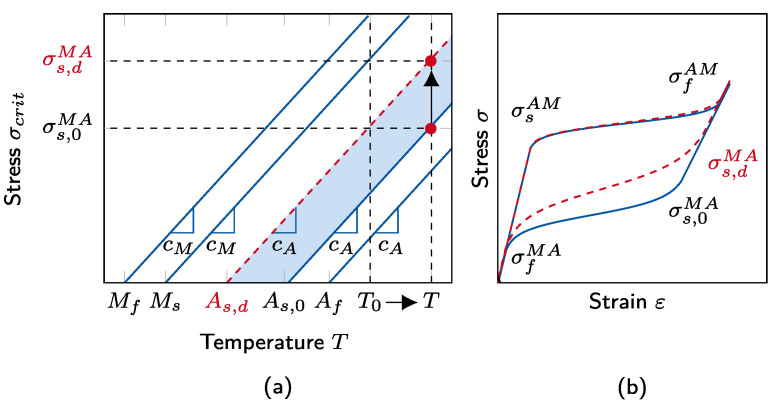
(**a**) The relation between the critical stress levels $\sigma_{crit}$ and the material temperature *T*. The austenite transformation start temperature $A_{s}$ is strain rate dependent. Here, $A_{s,0}$ and $A_{s,d}$ denote the austenite transformation start temperature for quasistatic and dynamic cases, respectively. (**b**) The corresponding stress–strain response of both quasistatic (solid line) and dynamic (dashed line) loading.

Moreover, four critical stress levels are defined to indicate the start and finish stresses of the phase transformations:

(7) $$\begin{array}{c} \begin{array}{cc} \sigma_{s}^{AM} & =c_{M}\left(T-M_{s}\right),\;\sigma_{f}^{AM}=c_{M}\left(T-M_{f}\right),\\ \sigma_{s,d}^{MA} & =c_{A}\left(T-A_{s,d}\right),\;\sigma_{f}^{MA}=c_{A}\left(T-A_{f}\right), \end{array} \end{array}$$

where $M_{s/f}$ and $A_{s/f}$ refer to the start/finish temperatures of martensite and austenite transformation, respectively, as shown in Figure 4. Here, $c_{M/A}$ are material constants indicating the critical stress–temperature slopes.

To model the dynamic response more accurately, in the formulation above, we use *T* as the material temperature and not as the environmental temperature, which was originally proposed by Zhu and Zhang, cf. [12]. In this way, the material model is able to consider both the quasistatic and dynamic cases, such that the critical stress levels are increased for high strain rates with increasing material temperatures. However, experiments show that a change in material temperature solely is not enough to initiate the *MA* transformation accurately. Hence, $A_{s,d}$ and $\sigma_{s,d}^{MA}$ are introduced, where the subscript ${}_{d}$ represents the modelling of the austenite transformation start temperature and the corresponding critical stress level with respect to dynamic effects.

This adaptation is also shown in Figure 4. Here, $T_{0}$ denotes the material temperature for the quasistatic load case. For dynamic load cases, the material temperature increases, such that a supplementary upward shift in the critical stress level is necessary to replicate the SMA response accurately. This behavior is achieved in the model by decreasing the austenite transformation start temperature from $A_{s,0}$ to $A_{s,d}$.

In the model, the evolution of the martensite fraction ξ is based on the Liang–Rogers model [33]. Its rate form is computed by

(8) $$\begin{array}{cc} A\to M:\; & \dot{\xi }=\left(1-\xi_{0}\right)\left(a_{M}\dot{T}-\frac{a_{M}}{c_{M}}\dot{\sigma }\right)g\left[a_{M}\left(T-T_{M}-\frac{\sigma }{c_{M}}\right)\right], \end{array}$$

(9) $$\begin{array}{cc} M\to A:\; & \dot{\xi }=\xi_{0}\left(a_{A}\dot{T}-\frac{a_{A}}{c_{A}}\dot{\sigma }\right)g\left[a_{A}\left(T-T_{A}-\frac{\sigma }{c_{A}}\right)\right], \end{array}$$

where $g=-e^{x}{\left(1+e^{x}\right)}^{-2}$ and

(10) $$T_{M}=\frac{M_{s}+M_{f}}{2},\;T_{A}=\frac{A_{s}+A_{f}}{2},\;a_{M}=\frac{\ln\left(10,000\right)}{M_{s}-M_{f}},\;a_{A}=\frac{\ln\left(10,000\right)}{A_{f}-A_{s}}$$

**Figure 5:**
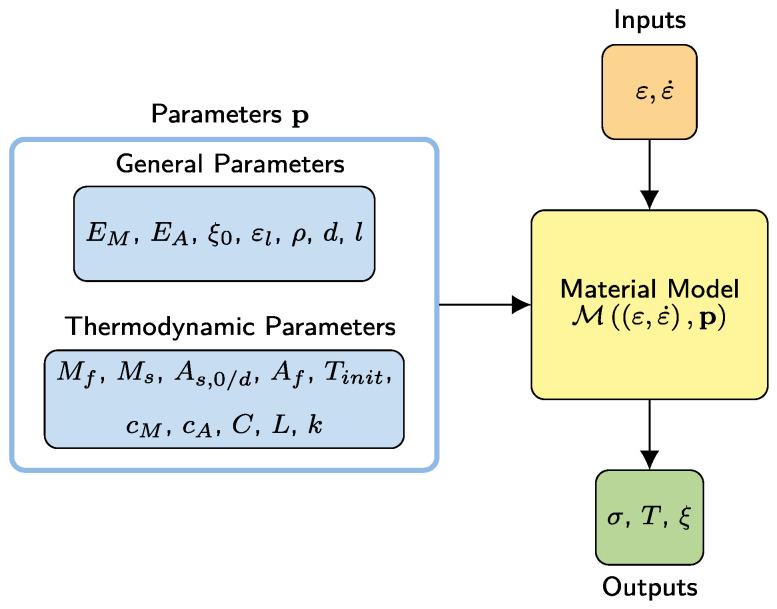
Input and output parameters of the material model. The constitutive model is driven by the strain ε and strain rate $\dot{\varepsilon }$ time histories. Stress σ, temperature *T*, and the martensite volume fraction ξ time histories are computed as model outputs.

Here, $\xi_{0}$ refers to the initial martensite fraction at the beginning of the current transformation. In the model of Zhu and Zhang [12], the outputs σ, *T*, ξ as well as their time derivatives, are solved simultaneously from the heat equation (Equation (5)), mechanical stress equation (Equation (6)), and the kinetic rules (Equations (8) and (9)) by time integration algorithms, such as the fourth-order Runge–Kutta method. Figure 5 illustrates the input and output parameters of the material model.

#### Parameter Influence on Stress–Strain Response

The parameters introduced in Section 2.3 have to be determined accurately as each of them affects the material model response. It is obvious that the Young’s moduli, $E_{M}$ and $E_{A}$, have a major influence on the stress–strain behavior. This effect can be easily seen in the elastic parts of the stress–strain response, where an unsuitable parameter would lead to inaccurate slopes in the σ-ε diagram. Furthermore, $\varepsilon_{l}$ denotes the maximum strain at ξ=1, such that it directly affects the strain level, where the phase transformation from austenite to martensite is finished, cf. Equation (4). In this study, purely superelastic material behavior is assumed since the ambient temperature is above the austenite transformation finish temperature ($T_{init}>A_{f}$). As a result, $\xi_{0}$ is assumed to be 0 in the initial austenite state. Nevertheless, this parameter is variable and changes as the martensite transformation proceeds. The ambient temperature $T_{init}$ has only a minor influence on the model response since the material temperature is used to compute the critical stress levels, cf. Equation (7). From Equation (6), it is obvious that the stress response increases for high specimen densities ρ. In addition, from Equation (5), it follows that greater specimen volumes *V*, or diameters *d*, lead to higher temperature evolutions. By contrast, high heat-transfer coefficients *k* result in greater heat transfer and thus in lower temperature evolutions. A major impact on the stress–strain response results from the critical stress levels given in Equation (7). These stress levels are influenced by the parameters $c_{M}$ and $c_{A}$, as well as $M_{f}$, $M_{s}$, $A_{s,d}$, and $A_{f}$. With increasing $c_{M}$ and $c_{A}$, stress levels also increase. Whereas greater transformation temperatures ($M_{f}$, $M_{s}$, $A_{s,d}$, $A_{f}$) lead to lower stress levels.

For the modelling of dynamic SMA response, particularly, the introduced dynamic austenite transformation start temperature $A_{s,d}$ has a high influence as with it an earlier reverse transformation can be started, cf. Figure 4. Furthermore, in case of high strain rates, the material temperature is increased due to self-heating effects. These effects are modelled by the specific and latent heat (*C* and *L*), which have the greatest influence on the dynamic model response as they are directly linked to the temperature evolution, cf. Equation (5). For the dynamic load case, finally, *k* is also important, which controls, as mentioned above, directly the heat transfer of the SMA.

## 3. Results and Discussion

### 3.1. Stress–Strain Experiments

**Figure 6:**
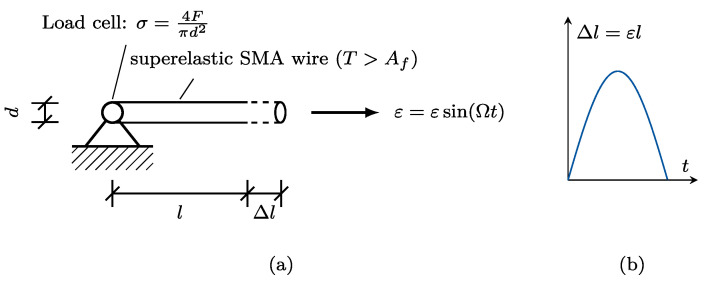
(**a**) Schematic representation of stress–strain experiments. The wire of diameter *d* and length *l* is fixed on one side and elongated by Δl. The resulting tensions and the applied strains are measured by a load cell and a laser position sensor, respectively. The ambient temperature is above the austenite transformation start temperature ($T>A_{f}$). (**b**) Applied displacement with respect to time.

For the generation of test data, cyclic tensile tests are conducted by a uniaxial shaking table, as schematically depicted in Figure 6. A pre-stress of $\sigma_{0}=134.9$ MPa was applied. The wire stress response was measured by a load cell. The applied strain was determined by a laser position sensor. Both sensors were sampled at 1000 Hz. The ambient temperature was around $T_{init}=296.2$ K (23.05 °C). In this study, a Ni-55.8%-Ti-43.95% SMA wire with l=150 mm length and d=0.2 mm diameter was investigated. For further information on the test setup, the reader is referred to [34]. All material parameters are reported in Table 1. Here, $E_{M}$, $E_{A}$, and $\varepsilon_{l}$ are determined via the stress–strain relation obtained under quasistatic load. Furthermore, ρ, $M_{f}$, $M_{s}$, $A_{s,0}$, and $A_{f}$ are provided by the manufacturer, whereas $c_{M}$ and $c_{A}$ are determined using Equation (7) from the provided parameters. Ambient temperature and geometric parameters (diameter and wire length) are measured before testing.

### 3.2. Identification of Thermodynamic Parameters

As introduced in Section 2.3, the constitutive SMA model of Zhu and Zhang relies on three thermodynamic parameters: the specific heat *C*, the latent heat *L*, and the heat-transfer coefficient *k*. All other thermodynamic parameters ($M_{f}$, $M_{s}$, $A_{f}$, $c_{M}$, and $c_{A}$) are generally provided by manufacturers (cf. Table 1) and can also be used in constitutive models with a sufficient accuracy for dynamic applications. Furthermore, $A_{s,0}$ is also provided, which is accurate enough for the quasistatic case and is now tuned as $A_{s,d}$ to model the dynamic response more accurately.

Accordingly, the searched parameter set reads:

(11) $$\begin{array}{c} p_{i}^{⊤}={\left[\begin{array}{c} C,\;L,\;k,\;A_{s,d} \end{array}\right]}_{i}\; \end{array}$$

where the parameter space of *C*, *L*, and *k* is determined based on their influence on the response of the constitutive model. For this purpose, the cyclic stress response of the SMA wire is computed by the material model for the strain amplitude ε=4% and the strain rate amplitude $\dot{\varepsilon }=50.27$% s^−1^. The parameters are alternated around the reference setting C=800 J(kgK)^−1^, L=8000 J kg^−1^, and k=0.021 W K^−1^, where the works of Kato [35] and Zhu and Zhang [12] are taken as reference. The chosen parameter space is listed in Table 2. The remaining material parameters that are used for this study, including $A_{s,d}=A_{s,0}$, correspond to Table 1.

**Figure 7:**
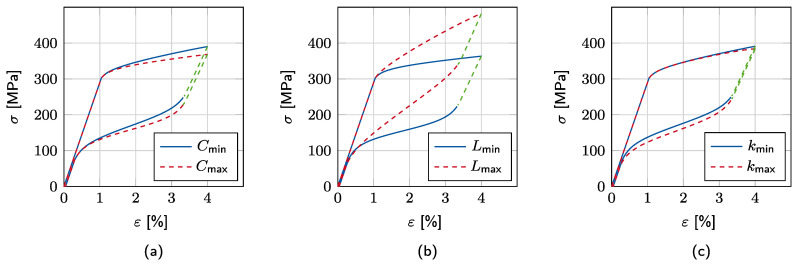
Influence of thermodynamic parameters on the model response: (**a**) Influence of specific heat *C*, (**b**) influence of latent heat *L*, and (**c**) influence of heat-transfer coefficient *k*. The green dashed line illustrates the linear elastic part of the unloading path.

The results are depicted in Figure 7. The smaller the specific heat *C*, the less energy is required to raise the material temperature. This results in greater temperature amplitudes and consequently leads to steeper transformation plateaus, as shown in Figure 7a. On the other hand, the latent heat provokes similar effects in the hysteresis of the stress–strain curve, as shown in Figure 7b. In fact, the latent heat is included in Equation (5) and thus directly linked to the temperature evolution. A high latent heat value results in an increase in temperature during the austenite-to-martensite phase transformation. A higher material temperature, in turn, leads to a more stable austenite phase, such that a higher mechanical stress is needed to proceed the phase transformation. Accordingly, *C* and *L* affect the model response oppositely and with different parameter combinations the same material response can be replicated. Finally, the higher the heat-transfer coefficient *k*, the more heat is transferred to the environment. Small *k* values thus lead to rising temperatures after each cycle as there is less heat transferred from the wire to the environment, as shown in Figure 7c.

Accordingly, the first three parameters of $p_{i}$ influence the slope of both forward- and reverse-phase transformations. However, these parameters do not affect the critical stress $\sigma^{MA}$ directly, which initiates the reverse transformation. As illustrated in Figure 7 by the green dashed lines, *C*, *L*, and *k* do not affect the length of the linear elastic martensite unloading path. This effect can be efficiently covered by alternating $A_{s}$. Its parameter space is also introduced in Table 2 and is determined similar to the prior ones considering the influence on the response of the constitutive model. Accordingly, $A_{s,d}$ is chosen, such that it covers the temperature range below $A_{s,0}$ until $M_{s}$, as described previously in Figure 4. It should be noted that the size of the parameter space does not have a major influence on the neural network accuracy as the best-fitting parameter *combination* is identified. On the other hand, with increasing parameter ranges, the required computational effort also increases. In this study, the ranges are chosen based on both numerical and experimental expertise from [12,34,35].

For the PI, a feedforward ANN is utilized with two hidden layers, where the first and second layers contain 128 and 64 neurons, respectively. The learning rate is initialized as $1\times 10^{-3}$. Training is completed after 50 epochs and is conducted with a batch size of 32. A Gaussian noise with zero mean and a variance of $1\times 10^{-4}$ is added to the training input data to make the network more stable to measurement errors. For the training of the ANN, i=2000 sets are sampled from the parameter space. The network architecture and its training parameters are determined after a parametric study by trial and error. Deeper network architectures lead to slightly more accurate predictions but at the cost of computational effort. Therefore, in this study, the simplest possible network architecture is chosen, which provides sufficient results. The generated data correspond to $D=\{\left(\sigma_{i},p_{i}\right),\forall i\in \left\{1:2000\right\}\}$, where 10% of them is used for validation. To consider strain rate effects, the data set is generated using the material model repeatedly for five different strain rate amplitudes of $\dot{\varepsilon }\in \left[1.26,\;2.51,\;12.57,\;25.13,\;50.27\right]$% s^−1^ with a constant strain amplitude of ε=4%. The generation of signal data and neural network training take less than 30 min using an AMD Ryzen 9 5950× CPU and computing on an NVIDIA GeForce RTX 3090, which is significantly lower than the experimental effort considering the time required for the specimen preparation, tests, and data processing.

After training the ANN, the σ-ε experiments are conducted, as described in Section 3.1 for the same strain rate and strain amplitudes. From the experimental stress–strain response, the parameters $\hat{C}$, $\hat{L}$, $\hat{k}$, and ${\hat{A}}_{s,d}$ are identified. Table 3 shows the estimated $\hat{p}$ after training with D. Here, the experimentally measured loading stress responses are divided into 200 equidistantly distributed points. The final values of the identified parameters are determined from the mean over all investigated strain rate cases, except for $A_{s,d}$, where only the dynamic load cases ($\dot{\varepsilon }\in \left[12.57,\;25.13,\;50.27\right]$% s^−1^) are considered.

**Figure 8:**
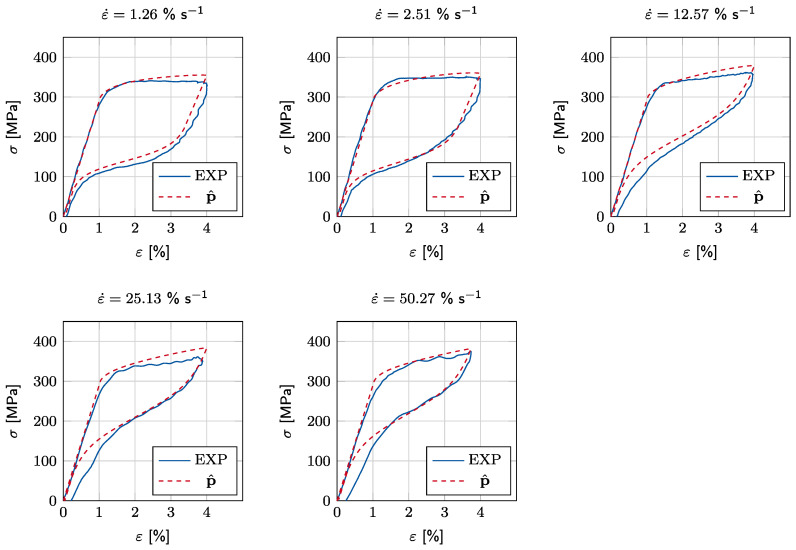
Comparison of the experimentally determined stress–strain responses (EXP) with numerical calculations, which use the identified parameter set $\hat{p}$.

Figure 8 compares the experimental results with the numerical calculations, which use the identified parameter set $\hat{p}$, where for the quasistatic cases ($\dot{\varepsilon }\in \left[1.26,2.51\right]$% s^−1^) the manufacturer provided $A_{s,0}$ is used in numerical calculations. The numerical calculations using $\hat{p}$ accurately match both the loading and unloading paths of experimental results. It is noteworthy that the accuracy of the numerical calculations is limited by the capability of the constitutive model. Therefore, the effects, such as residual deformations and hardening, are not replicated accurately. We observe from the experimental results that with an increasing strain rate, the residual wire strain becomes significantly visible. This effect is currently not replicated by the model. With these results, we would like to emphasize that the accuracy of the proposed PI is limited by the constitutive model precision. The effects that are not included in the basis material model cannot be considered in the PI process.

**Figure 9:**
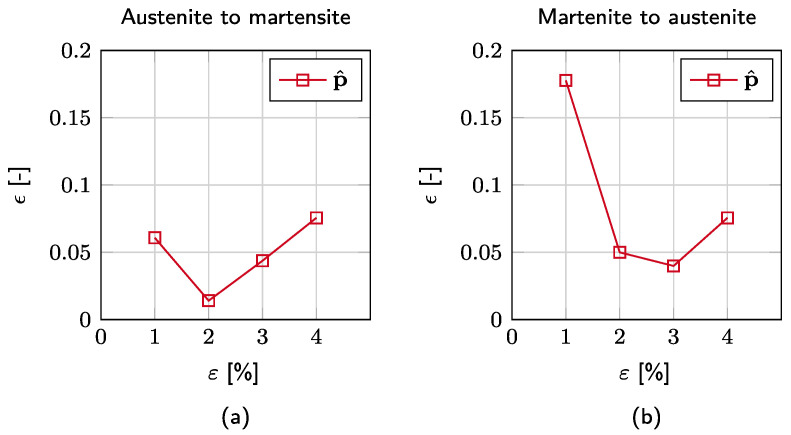
Mean value of the relative cumulative stress error corresponding to the used parameter set $\hat{p}$ for both forward (**a**) and reverse (**b**) transformations.

From the results, the mean value of the relative cumulative stress error $\epsilon_{n}$ is computed as

(12) $$\epsilon_{n}=\sum_{m}^{5}\left(\frac{\left|\Delta \sigma_{m,n}\right|}{\sigma_{\mathrm{EXP},m,n}}\right)/5$$

where $\Delta \sigma_{m,n}$ is the difference between the estimated and experimentally determined stress $\sigma_{\mathrm{EXP},m,n}$ of the corresponding strain intervals n∈[1,2,3,4]% for both forward and reverse transformations. Here, the mean is computed over m cases corresponding to the number of investigated strain rate amplitudes. As shown in Figure 9, the error value is located around 5%, neglecting the value for 1% strain on the reverse transformation. In this case, the error value is high, which is due to the material model’s inability of modelling residual deformations, cf. Figure 8.

## 4. Conclusions

In the present paper, for the identification of thermodynamic parameters of superelastic SMA wires, an ANN-based PI methodology is presented. The proposed approach was coupled with a macroscopic constitutive model. Here, to consider strain-rate-dependent response effects more accurately, two austenite transformation start temperatures were defined distinguishing between the quasistatic and dynamic cases. Furthermore, in the model, the current material temperature was considered for a strain rate sensitive calculation of critical stress levels. In an initial step, training data were generated by the constitutive model. Here, the searched thermodynamic parameters were sampled from a parameter space, and for each sampling, the stress responses were computed. After training, the ANN estimated the searched parameters from conventional stress–strain experiments. Finally, to validate the accuracy of the proposed method, numerical simulations were conducted, and results were compared with experimental data.

## Figures and Tables

**Table 1:** Constitutive model parameters of the SMA wires used in this study.

Parameter		Value	Unit
Young’s modulus of martensite	$E_{M}$	14,100	MPa
Young’s modulus of austenite	$E_{A}$	29,000	MPa
Critical stress–temperature slope of martensite	$c_{M}$	6.13	MPa K^−1^
Critical stress–temperature slope of austenite	$c_{A}$	6.57	MPa K^−1^
Maximum strain at ξ=1	$\varepsilon_{l}$	0.04	-
Martensite transformation finish temperature	$M_{f}$	231.4	K
Martensite transformation start temperature	$M_{s}$	247.7	K
Austenite transformation start temperature	$A_{s,0}$	263.2	K
Austenite transformation finish temperature	$A_{f}$	285.2	K
Ambient temperature	$T_{init}$	296.2	K
Density	ρ	6500	kg m^−3^
Diameter	*d*	0.0002	m
Wire length	*l*	0.15	m

**Table 2:** Parameter space of the searched thermodynamic parameters $p_{i}$.

Parameter		min	max	Unit
Specific heat	*C*	800	2000	J(kgK)^−1^
Latent heat	*L*	400	40,000	J kg^−1^
Heat-transfer coefficient	*k*	0.001	0.100	W K^−1^
Austenite transformation start temperature	$A_{s,d}$	247.0	263.2	K

**Table 3:** Thermodynamic parameter set $\hat{p}$ identified by the ANN after training with the data set D.

$\dot{\varepsilon }$ [% s^−1^]	ε [%]	$\hat{C}$ [J(kgK) ^−1^]	$\hat{L}$ [J kg^−1^]	$\hat{k}$ [W K^−1^]	${\hat{A}}_{s,d}$ [K]
1.26	4	1146	5404	0.084	$A_{s,0}$
2.51	4	1824	18,773	0.089	$A_{s,0}$
12.57	4	1827	18,583	0.090	247.3
25.13	4	1888	23,278	0.089	247.4
50.27	4	1896	25,108	0.089	248.0
Final result $\hat{p}$:		1716	18,229	0.088	247.6

## Data Availability

The data presented in this article are available on request from the corresponding author.

## Abbreviations

The following abbreviations are used in this manuscript:

ANN	Artificial Neural Network
LHS	Latin Hypercube Sampling
SMA	Shape Memory Alloy
AM	Austenite to Martensite
MA	Martensite to Austenite
ML	Machine Learning
PI	Parameter Identification
